# Integrated Transcriptomic and Metabolic Framework for Carbon Metabolism and Plant Hormones Regulation in *Vigna radiata* during Post-Germination Seedling Growth

**DOI:** 10.1038/s41598-020-60771-3

**Published:** 2020-02-28

**Authors:** Hong Wang, Xinbo Guo, Quan Li, Yanyan Lu, Wenjie Huang, Fangyuan Zhang, Ling Chen, Rui Hai Liu, Shijuan Yan

**Affiliations:** 1 School of Food Science and Engineering, Ministry of Education Engineering Research Centre of Starch & Protein Processing, Guangdong Province Key Laboratory for Green Processing of Natural Products and Product Safety, South China University of Technology, Guangzhou, 510640 China; 2 Agro-biological Gene Research Center, Guangdong Academy of Agricultural Sciences, Guangzhou, 510640 China; 3 School of Life Science, Southwest University, Chongqing, 400715 China; 4 Department of Food Science, Stocking Hall, Cornell University, Ithaca, NY 14853 USA

**Keywords:** Plant hormones, Transcriptomics, Metabolomics

## Abstract

During mung bean post-germination seedling growth, various metabolic and physiological changes occurred, leading to the improvement of its nutritional values. Here, transcriptomic and metabolomic analyses of mung bean samples from 6-hour, 3-day and 6-day after imbibition (6-HAI, 3-DAI, and 6-DAI) were performed to characterize the regulatory mechanism of the primary metabolites during the post-germination seedling growth. From 6-HAI to 3-DAI, rapid changes in transcript level occurred, including starch and sucrose metabolism, glycolysis, citrate cycle, amino acids synthesis, and plant hormones regulation. Later changes in the metabolites, including carbohydrates and amino acids, appeared to be driven by increases in transcript levels. During this process, most amino acids and monosaccharides kept increasing, and accumulated in 6-day germinated sprouts. These processes were also accompanied with changes in hormones including abscisic acid, gibberellin, jasmonic acid, indole-3-acetic acid, etc. Overall, these results will provide insights into molecular mechanisms underlying the primary metabolic regulation in mung bean during post-germination seedling growth.

## Introduction

Mung bean (*Vigna radiata*), a protein-rich leguminous food crop with short growth cycle (70–90 days), is cultivated in China, India, Southeast Asia, Central Africa, and North America. As a nutritional and healthy ingredient, mung bean is widely consumed in cuisine, such as soups and congee, in cakes and noodles, and in miscellaneous snacks. It is also used to alleviate heat stress and regulate gastrointestinal upset in traditional medicine^1^. Recent studies have explored its diversified bioactivities. For example, mung bean protein has been proved to prevent non-alcoholic fatty liver disease in high-fat fed mice^2^. Hypoglycaemic activity has been reported in mung bean coat and extract in rodents^3,4^, which might be contributed by inositol and phenolic content^5,6^. The nutritional and medicinal qualities of the mung bean is enhanced by germination due to a spectrum of significant changes in metabolites^1^.

Germination is a complex process with various metabolic and physiological changes, which commences with imbibition and terminated with the elongation of the embryonic axis^7^. During germination, macromolecular substances such as polysaccharides, proteins and lipid stored in the seeds are degraded into small active compounds, accompanied with energy production. Studies have demonstrated that this process was regulated by the interaction of phytohormones, such as abscisic acid (ABA), gibberellin (GA), jasmonic acid (JA), and indole-3-acetic acid (IAA), etc^8–10^.

Recent studies have shown that mung bean sprouts offer more free amino acids, organic acids, monosaccharides, vitamins, and phytochemicals compared with mung bean^1,11–13^. The variation in metabolites during mung bean germination is accompanied with dynamic regulation at transcription level. The results of genome sequencing fuelled several transcriptome projects comprising multiple developmental stages and environmental conditions. Over the past 10 years, several legume species have released their own genomes, including soybean (*Glycine max*)^14^, mung bean (*Vigna radiata*)^15–17^, common bean (*Phaseolus vulgaris*)^18^, chickpea (*Cicer arietinum*)^19^, and diploid ancestors of cultivated peanut^20^. Moreover, the transcriptomic and metabolomic analysis have been performed on soybean during germination, respectively^21,22^, shedding light on the regulation and nutritional change during germination. Nevertheless, the global transcriptional patterns of mung bean seedlings during germination remain poorly known, which might limit the understanding of the molecular mechanisms underlying the regulation of metabolism during mung bean germination and therefore application awaits further study. Therefore, the objective of this study was to integrate transcriptome and metabolite profiles of mung bean and seedlings at 6-hour, 3-day and 6-day after imbibition (6-HAI, 3-DAI and 6-DAI), which would provide a systematic view of mung bean response to germination. Together, the study would expand the understanding and application of germination to enhance nutritional qualities in sprouts.

## Results

### Transcriptome sequencing during mung bean post-germination seedling growth

**Figure 1:**
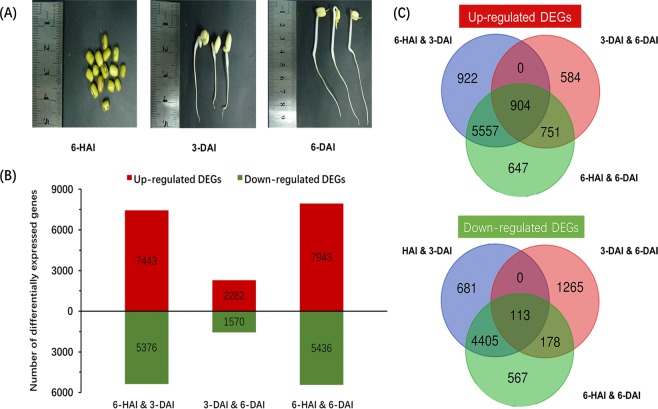
General profile of mung bean during post-germination seedling growth. (**A**) Photographs of mung bean during post-germination seedling growth. (**B**) Comparison of the numbers of significantly up-regulated (red) and down-regulated (green) genes between two growth time points (6-HAI, 3-DAI and 6-DAI). (**C**) Venn diagrams of up- and down-regulated significantly genes detected among three growth time points.

Mung bean germination occurred upon imbibition, with penetration of the coat surrounding the embryo, growth of the embryonic axis from 6-HAI to 3-DAI (Fig. 1A). Subsequently the embryonic axis was further elongated, in addition, the leave and root of mung bean sprouts were observed (Fig. 1A). To understand the transcriptional landscape of mung bean post-germination seedling growth, three biological replicates from 6-hour, 3-day and 6-day after imbibition were subjected to RNA extraction and transcriptome sequencing (termed as 6-HAI, 3-DAI and 6-DAI, respectively). After raw quality filtering, a total of 43.02 Gb of clean sequence data (34 to 41 million clean reads per sample) were generated from nine samples (Table S1), which was comparable to that produced by a recent mung bean deep RNA-sequencing study^15^. The percentage of reads successfully mapped on the mung bean reference genome was 78.7–83.9%, and 77.4–82.7% of reads were uniquely mapped (Table S1). To comprehensively identify new genes and refine the existing gene models, 1163 new genes were assembled after filtering the transcript with less than 50 amino acids or one exon. Approximately 812 new genes were annotated based on five main databases, including Gene ontology (GO), KEGG Orthology (KO), Swiss-Prot protein (Swiss-Prot), EggNOG, NCBI non-redundant protein (NR), which will enrich the database of mung bean transcriptome (Table S2).

### Distinct gene expression pattern for the three seedling growth stages of post-germination

The process of mung bean germination yielded substantial changes in gene expression (Table S3). High correlation among the three biological replicates were shown in the heatmap, indicating high degree of reproducibility with an average Pearson’s correlation coefficient of r^2^ ≥ 0.95 (Fig. S1A). Globally, the correlation coefficiencies between 3-DAI and 6-DAI were over 0.70, whereas the correlation coefficiencies between 6-HAI and others were below 0.04, indicating more significant changes were observed between seeds and seedlings. Furthermore, heatmap clustering on expressed genes showed that the mRNA population compromised two distinct subpopulations: transcripts higher expressed in initial germination stage (6-HAI) following by a decreased and stable trend, and transcripts lower expressed in 6-HAI and increased at 3-DAI or 6-DAI (Fig. S1B).

To understand the gene expression differences among the three different stages during post-germination seedling growth, differentially expressed genes (DEGs) were identified by applying cutoff of |log_2_ fold change| > 1 and FDR < 0.01(Table S3). The striking changes in transcription levels occurred between 6-HAI and other two time points. 12819 and 13379 DEGs were identified in 6-HAI vs. 3-DAI and 6-HAI vs. 6-DAI, respectively, while 3852 DEGs were observed between 3-DAI and 6-DAI. There were 7943 up-regulated and 5436 down-regulated transcripts between 6-HAI and 6-DAI, while 2282 up-regulated and 1570 down-regulated transcripts between 3-DAI and 6-DAI (Fig. 1B). These results were associated with the lower correlation coefficiencies between 6-HAI and other samples in heatmap plot (Fig. S1A).

The unique and common DEGs during post-germination seedling growth were shown by the Venn diagram (Fig. 1C). There was a core set of DEGs among the three growth periods (904 up-regulated and 113 down-regulated), which likely revealed sustainable response to post-germination growth at transcript level. Additionally, there were no specifically overlap of DEGs between 3-DAI and other two samples, whereas most up- or down-regulated DEGs were specially shared between two mung bean sprouts and 6-HAI, suggesting substantial changes occurred in the 3-DAI mung bean sprouts.

**Figure 2:**
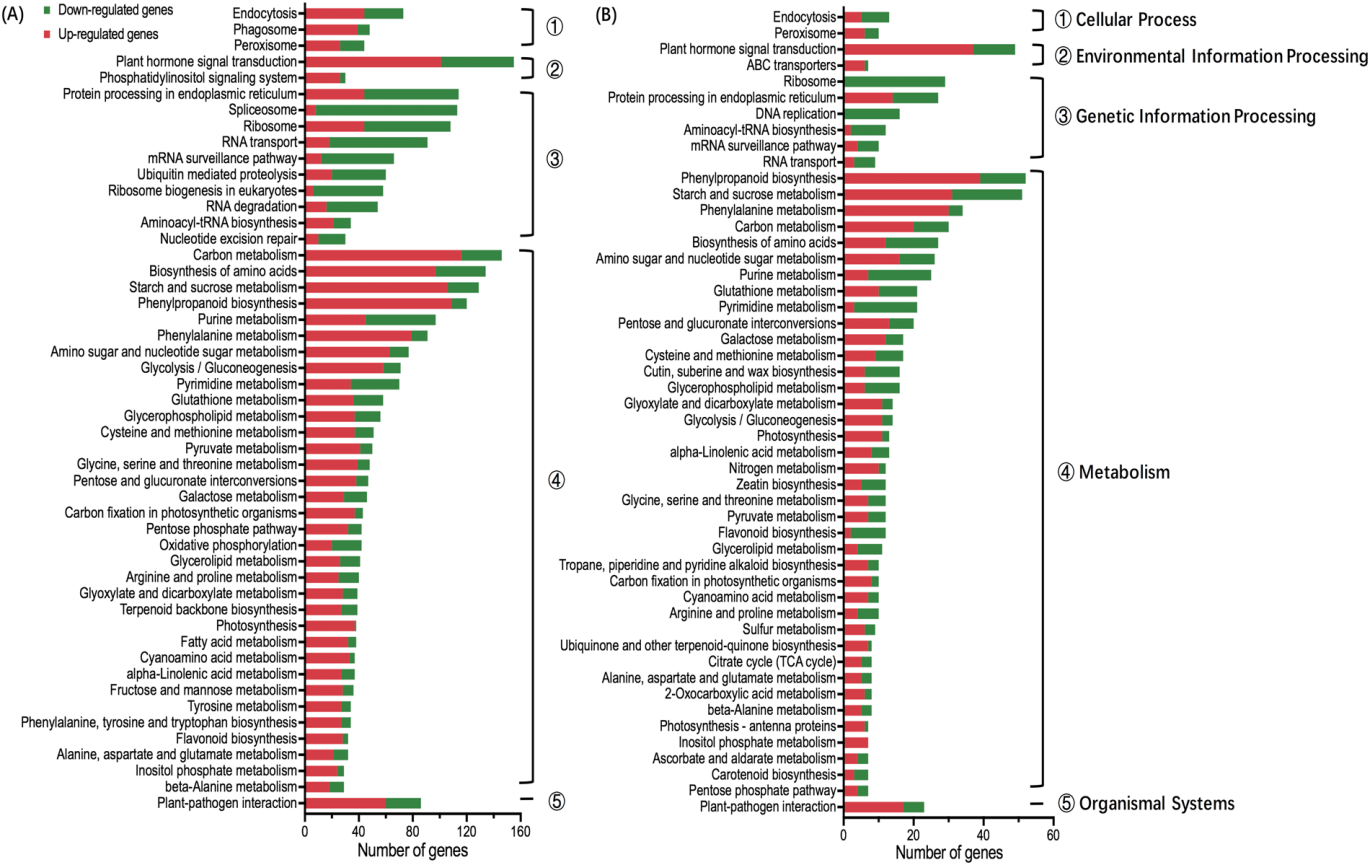
KEGG classification of DEGs between sequential growth time points. (**A**) 6-HAI vs. 3-DAI and (**B**) 3-DAI vs. 6-DAI presented the top 50 KEGG pathways with the most abundance of DEGs, respectively. Regulatory profiles were presented as the number of up-regulated (red) and downregulated genes (green), within each category of KEGG pathways. All the pathways were classified into five categories, including cellular process, environmental information processing, genetic information processing, metabolism, and organismal systems.

To place these substantial changes in gene expression into a meaningful context, DEGs were further classified based on KEGG pathway. A total of 2456 DEGs and 645 DEGs were annotated to 124 and 111 pathway annotations in 6-HAI vs. 3-DAI and 3-DAI vs. 6-DAI, respectively (Table S4). All the pathways were classified into five categories, including cellular process, environmental information processing, genetic information processing, metabolism, and organismal systems and the most abundance of DEGs were identified in the pathway belonged to metabolism (Fig. 2, Table S4). The pathways with the greatest representation of annotated DEGs included plant hormone signal transduction, carbon metabolism, biosynthesis of amino acids, starch and sucrose metabolism, and phenylpropanoid biosynthesis in both 6-HAI vs. 3-DAI and 3-DAI vs. 6-DAI. It was noteworthy that, at the early growth stage (Fig. 2A), more DEGs were upregulated in the cellular process, environmental information processing, metabolism, and organismal systems, whereas more DEGs allocated into pathways involved in genetic information processing were inhibited. At the later growth stage, the number of DEGs was decreased, so was the number of pathways containing more up-regulated DEGs (Fig. 2B).

The data demonstrated that the major biochemical differences during the post-germination seedling growth were reflected in part by highly dynamic transitions in transcriptome abundance, which might initiate shifts in metabolite pools in mung bean during post-germination seedling growth stages.

### Metabolome differences between 6-HAI, 3-DAI and 6-DAI growth stages

To validate the hypothesis mentioned above, metabolites profiling of 6-HAI, 3-DAI and 6-DAI mung bean samples were performed using a gas chromatography tandem mass spectrometry (GC-MS)-based metabolomics approach. After pre-processing the detected signals, a total of 160 metabolite features were detected. 60 metabolite peaks (representing 57 metabolites) were conclusively identified based on their authentic standards (Table S5). Among the 57 identified metabolites, there were 14 sugar metabolism-related compounds, 23 amino acid metabolism-related compounds, 15 tricarboxylic acid (TCA) and other organic acid metabolism-related compounds, and 5 other compounds. Most of the identified metabolites were associated with primary metabolism.

**Figure 3:**
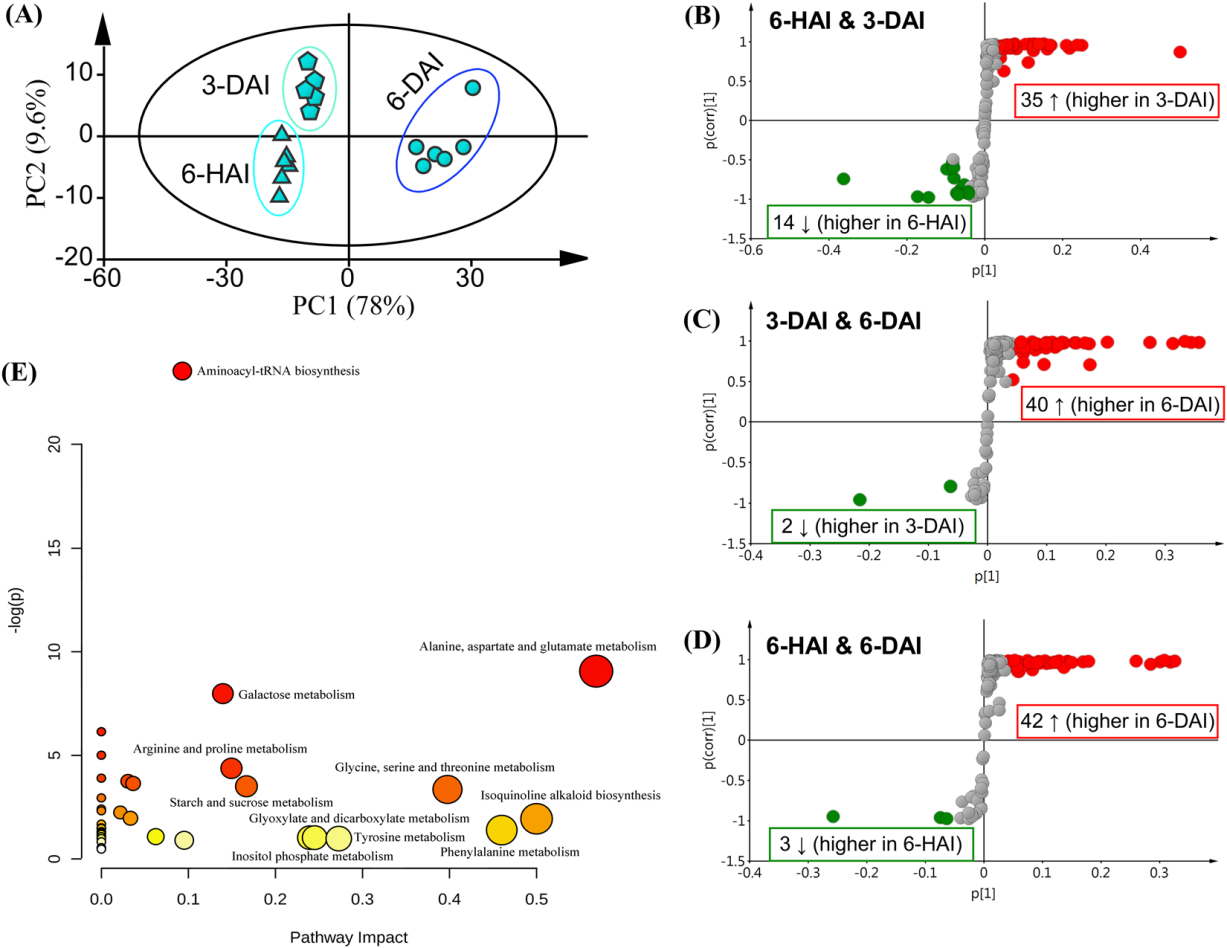
Multivariate statistical analysis of the untargeted metabolomic data from GC-MS. (**A**) The score plot generated from GC-MS data using the PCA model, demonstrating the dynamic metabolome change for the three groups including 3-DAI, 6-DAI and 6-HAI. (**B–D**) The S-plot generated from the OPLS-DA model with the pairwise comparison between (**B**) 6-HAI and 3-DAI, (**C**) 3-DAI and 6-DAI, and (**D**) 6-HAI and 6-DAI. Red or green-marking circles in S-plot are model-separated metabolites based on a variable importance of projection (VIP) > 0.5 and |p(corr)| ≥ 0.5. The up arrow indicated metabolites with the increased level for each pairwise comparison, and the down arrow indicated the opposite trend. (E) The pathway analysis of the model-separated metabolites indicated remarkable differences among 3-DAI, 6-DAI and 6-HAI groups. The x-axis represented the pathway impact, and y-axis represented the pathway enrichment. Larger sizes and darker colors represented higher pathway enrichment and higher pathway impact values, respectively.

To further compare the metabolome differences between the three groups, multivariate statistical analysis was conducted. Firstly, the score–plot of PCA revealed that samples of the 6-HAI, 3-DAI and 6-DAI groups were clustered well (Fig. 3A), and the 3-DAI and 6-DAI groups were more clearly distinguished from the 6-HAI group respectively (Fig. 3A). Parameters for evaluating the predictive ability and fitting level of the models showed an extremely satisfactory fit with good predictive power: R^2^Y and Q2 were both greater than 0.9. After model diagnosis, S-plot analyses allowed us to identify metabolites that can discriminate the two compared groups. Based on variable importance of projection (VIP) scores > 0.5 and |P(corr)| ≥ 0.5, a total of 49, 42 and 45 metabolites were selected and labelled as biomarkers for 6-HAI vs. 3-DAI, 3-DAI vs. 6-DAI and 6-HAI vs. 6-DAI, respectively (Fig. 3B–D). Both compared with the first growth point (6-HAI), 35 out of 49 metabolites were increased in abundance at 3-DAI (Fig. 3B), and 42 out of 45 metabolites were increased in abundance at 6-DAI (Fig. 3D). Among them, the contents of 29 metabolites kept being increased from 6-HAI to 6-DAI (Table S5).

The metabolome view map generated by the software Metaboanalyst 3.0 revealed that 5 relevant pathways were significantly enriched for those 57 metabolites based on P-value (<0.05) and impact value (>0.1) (Fig. 3E). The impact values for those 5 pathways, all of which involved to primary metabolism including alanine, aspartate, glutamate metabolism, galactose metabolism, arginine and proline metabolism, glycine, serine and threonine metabolism, and starch and sucrose metabolism, were 0.569, 0.140, 0.150, 0.398, and 0.167, respectively (Fig. 3E).

### Carbohydrate metabolism during post-germination seedling growth

**Figure 4:**
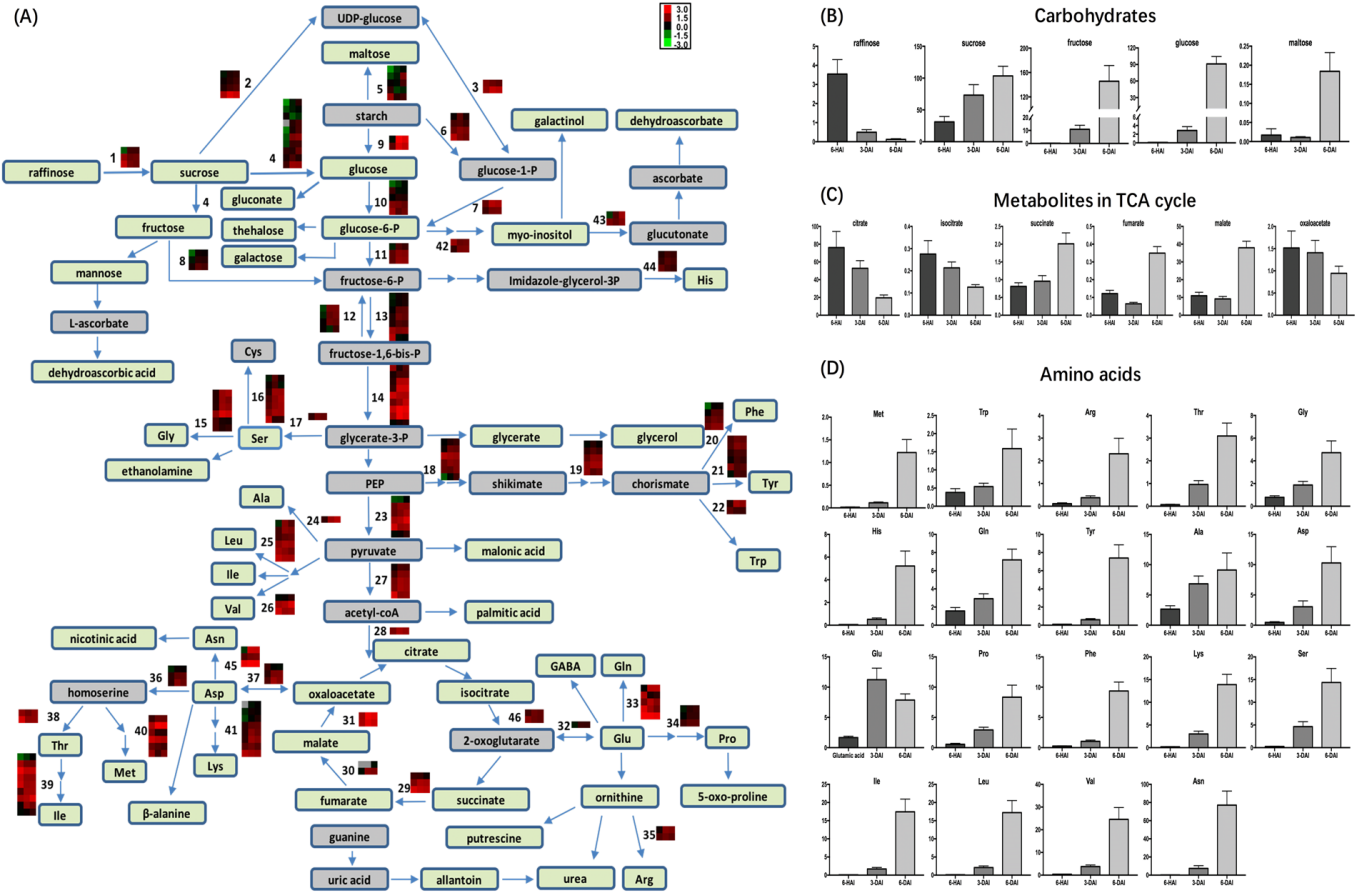
Schematic of differentially expressed transcripts and identified metabolites associated with primary metabolism. (**A**) The DEGs involved in starch-sucrose metabolism, glycolysis, TCA cycle, and biosynthesis of amino acids during mung bean post-germination seedling growth. Transcript level of DEGs encoding enzymes involving metabolic conversions were expressed as log_10_FPKM in the heat maps. The three columns in each heat map represented the growth time points (6-HAI, 3-DAI and 6-DAI). The grey blocks indicated the transcript level was not quantified due to low expression. These DEGs were detailly exhibited in Table S6. The metabolites in the green box were detected by GC-MS, the grey ones represented undetected. (**B–D**) The relative content of identified (**B**) carbohydrates, (**C**) metabolites involved in TCA cycle, and (**D**) amino acids with significant differences were shown in the histograms. The horizontal axis of the bars indicated the normalized intensity.

The differential expression transcripts and metabolite profiles concerning carbohydrate metabolism during mung bean post-germination seedling growth were presented in Fig. 4 and Table S6. The changes of DEGs encoding the enzymes involved in starch and sucrose metabolism mostly occurred at the early stage of growth (6-HAI to 3-DAI), and then maintained stable, indicating the corresponding changes occurred in sugar content (Figs. 4A,B). Sucrose was the major soluble carbohydrate in 6-HAI, which increased significantly from 6-HAI to 6-DAI. During the post-germination seedling growth, raffinose concentration sharply decreased, whereas fructose and glucose showed significantly increased trends (Fig. 4B).

The monosaccharides subsequently enter into the glycolysis and TCA cycle, which were also associated with early activation (6-HAI to 3-DAI) of genes, including 3 fructokinase DEGs, 1 out of 5 hexokinase DEGs, 3 out of 5 fructose-1,6-bisphosphatase DEGs, 4 out of 7 6-phosphofructokinase DEGs, 4 glyceraldehyde-3-phosphate dehydrogenase DEGs, 3 out of 6 pyruvate kinase DEGs, 4 pyruvate dehydrogenase DEGs, 2 isocitrate dehydrogenase DEGs, 1 out of 3 succinate dehydrogenase DEGs and 1 out of 2 fumarate hydratase DEGs (Fig. 4A, Table S6). However, the concentration of metabolites involved in the TCA cycle showed various changes (Fig. 4C). The levels of citrate and isocitrate decreased time-dependently, remaining less than one fourth and one half of the initial contents, respectively. Whereas succinate, fumarate and malate significantly increased about 2.5, 2.9 and 3.5 times in 6-day germinating mung bean sprouts, which were also accompanied with up-regulation of 7 DEGs mapping to succinate dehydrogenase, fumarate hydratase and malate dehydrogenase.

### Amino acid accumulation during post-germination seedling growth

In general, most of amino acids (19 of 20 essential amino acids) detected in the present study varied markedly during post-germination seedling growth (Fig. 4D). The higher contents of amino acids in mung bean at 6-HAI were alanine and glutamic acid. All the essential amino acid contents were potentially increased with growth, although the levels of methionine, glutamine and tyrosine were comparably lower in mung bean sprouts at 6-HAI. Intriguingly, asparagine became the most abundant amino acid in 6-day sprouts (Fig. 4D).

The amino acid accumulation was also associated with induction at transcripts level (Fig. 4A, Table S6). Among 84 DEGs annotating enzymes in biosynthesis pathway of amino acids (Table S6), about 60% of them were up-regulated and 24% of them were down-regulated at the early stage of mung bean seedling growth, however, less significant changes were observed at the later stage of seedling growth (11 up-regulated vs. 9 down-regulated DEGs).

### Phytohormone metabolism and signalling transduction during post-germination seedling growth

**Figure 5:**
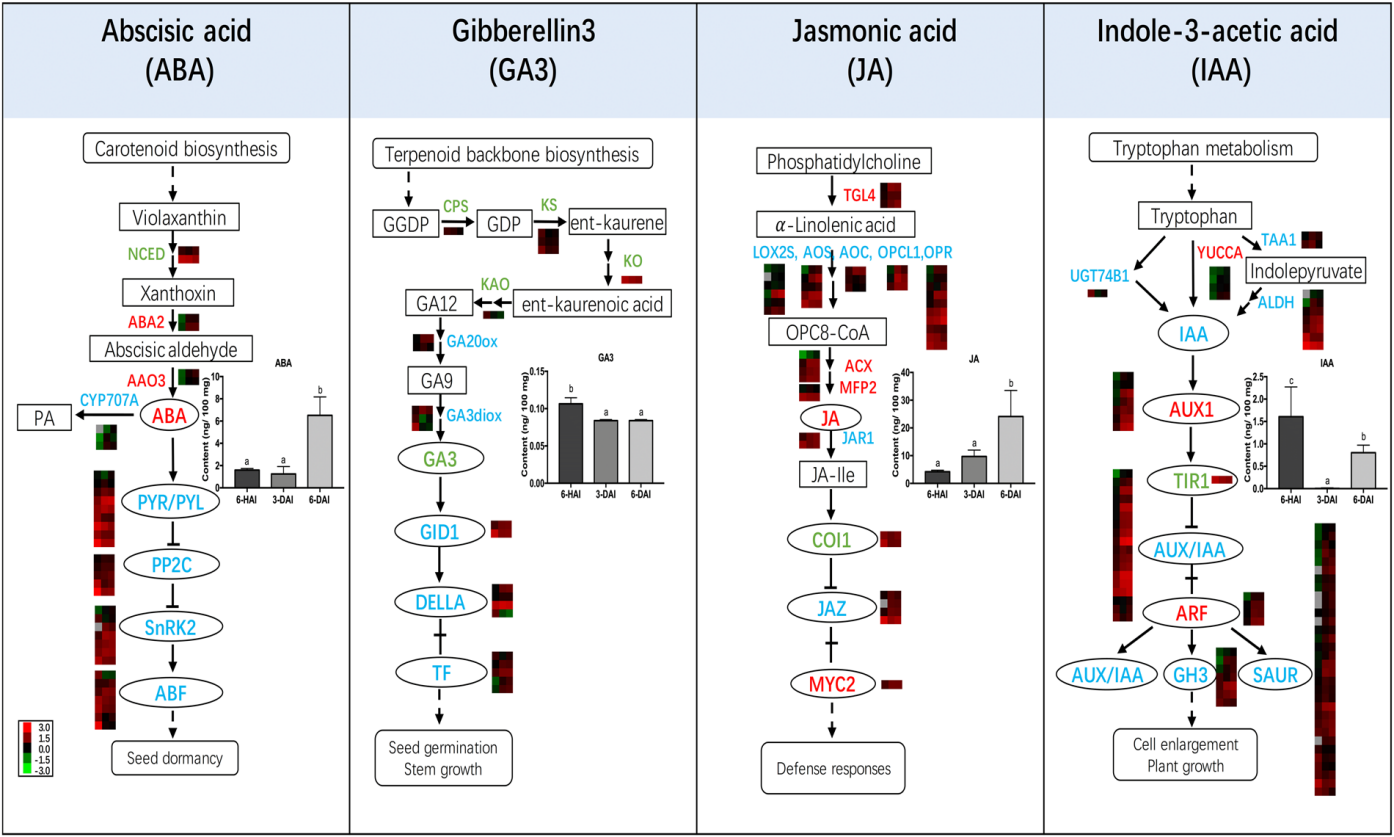
Expression pattern of DEGs and profile changes of phytohormones during mung bean post-germination seedling growth. Key enzymes and proteins were presented as their names (in red up-regulated, in green down-regulated, in blue with mixed up- and down-regulated DEGs). Transcript abundance of DEGs were expressed as log_10_FPKM in heat maps. The three columns in each heat map represented the growth time points (6-HAI, 3-DAI and 6-DAI). The grey blocks indicated the transcript level was not quantified due to low expression. These DEGs were detailly exhibited in Table S8. The changes of related phytohormone contents were visualized on the bar graphs. Bars with different letters differed significantly at p < 0.05.

Seedling growth is mainly regulated by the concerted interaction of diverse endogenous plant hormones (Fig. 5, Table S7). During the post-germination seedling growth, the contents of ABA and JA were significantly accumulated in 6-day mung bean sprouts, which increased from 1.59 ± 0.15 (6-HAI) to 6.51 ± 1.66 ng/100 mg (6-DAI) and from 4.23 ± 0.52 (6-HAI) to 24.12 ± 9.39 ng/100 mg (6-DAI), respectively. As to gibberellin A3 (GA3) and IAA, the highest contents were both observed at 6-HAI, with the value of 0.107 ± 0.008 and 1.61 ± 0.66 ng/100 mg, respectively. Then, GA3 content was reduced about 21.2% at 3-DAI and maintained stable at 6-DAI. As to IAA, a dramatic decrease was found at 3-DAI but increased again to 0.803 ± 0.169 ng/ 100 mg at 6-DAI (Fig. 5).

The levels of these phytohormones were regulated by their biosynthesis and catabolism (Fig. 5, Table S8). Of the several steps involved in the biosynthesis of ABA, 2 DEGs encoding 9-cis-epoxycarotenoid dioxygenase (NCED) were down-regulated at the two germinating stages, respectively; whereas 4 DEGs mapping to xanthoxin dehydrogenase (ABA2) and abscisic-aldehyde oxidase (AAO3) were upregulated from 6-HAI to 3-DAI. At the same germinating stage, 2 DEGs encoding abscisic acid 8’-hydroxylase (CYP707A), the key enzyme involved in ABA catabolism, were also up-regulated. As for GAs, more DEGs (7 down-regulated vs. 3 up-regulated) involved in *de novo* synthesis pathway were decreased from 6-HAI to 3-DAI, which were positively associated with a decrease in GA3 at this stage. Similar positive association was also observed in the changes of JA amounts and the DEGs involved in its biosynthesis pathways. 3 phospholipase A2 (TL4) DEGs, 4 out of 5 lipoxygenase (LOX2S) DEGs, 3 out of 4 hydroperoxide dehydratase (AOS) DEGs, 2 allene oxide cyclase (AOC) DEGs, 4 out of 10 12-oxophytodienoic acid reductase (OPR) DEGs, 3 acyl-CoA oxidase (ACX) DEGs, and 1 enoyl-CoA hydratase/3-hydroxyacyl-CoA dehydrogenase (MFP2) DEG were activated from 6-HAI to 3-DAI, and 7 out of 9 DEGs kept increasing trends till 6-DAI.

Generally, the expression patterns of DEGs involved in phytohormones signal transduction were evidently different. There were 151 and 49 hormone signal DEGs being observed during the early and later seedling growth stages, respectively (Table S8), including ABA signalling (24 vs. 7), GA signalling (11 vs.2), JA signalling (9 vs. 0), IAA signalling (57 vs. 19), cytokinin signalling (13 vs.5), brassinosteroid signalling (17 vs. 5), ethylene signalling (10 vs.3) and salicylic acid signalling (10 vs.8). From 6-HAI to 3-DAI, the amounts of up-regulated DEGs related to GA (8), JA (5), IAA (47), cytokinin (6), and brassinosteroid (13) signalling were greater than those of down-regulated DEGs, while the hormone signalling encoded by more up-regulated DEGs included ABA (4), GA (2), IAA (15), cytokinin (3), ethylene (3) and salicylic acid (8) from 3-DAI to 6-DAI. Furthermore, the numbers of auxin (IAA) signalling genes involved in cell enlargement and plant growth exceeded the numbers of DEGs related to other hormones signalling. Among these components of IAA signalling, DEGs mapping to auxin influx carrier (AUX1) and auxin response factor (ARF) showed increasing trends at both two growth stages, whereas transport inhibitor response 1 (TIR1) DEG was decreased during mung bean post-germination seedling growth.

### Quantitative real-time PCR (qRT-PCR) validation

19 candidate genes were selected to validate the results of transcriptomic sequencing. Among these genes, 14 genes were up-regulated and 5 genes were down-regulated between 6-HAI and 3-DAI, while 13 genes were up-regulated and 6 genes were down-regulated between 3-DAI and 6-DAI (Table 1). Generally, these gene dynamic changes from the qPCR were consistent to those from the RNA-sequencing results. These results suggested the applicability of RNA-sequencing to mung bean transcriptome analysis is a reliable way to find DEGs during post-germination seedling growth.

**Table 1:** Comparison of transcript expression for selected genes as measured by RNA sequencing and qRT-PCR.

ID	RNA sequencing		qPCR	
	6-HAI vs. 3-DAI	6-HAI vs. 6-DAI	6-HAI vs. 3-DAI	6-HAI vs. 6-DAI
LOC106767203	0.403^*^	0.897	0.429 ± 0.041^*^	0.246 ± 0.034^*^
LOC106753951	0.047^*^	0.341^*^	0.037 ± 0.007^*^	0.027 ± 0.005^*^
LOC106760472	16.846^*^	54.976^*^	2.887 ± 0.212^*^	1.603 ± 0.160^*^
LOC106754175	45.186^*^	102.854^*^	3.274 ± 0.261^*^	4.295 ± 0.589^*^
LOC106772114	50.572^*^	149.266^*^	8.097 ± 1.026^*^	4.100 ± 0.889^*^
LOC106755252	114.279^*^	248.152^*^	42.926 ± 5.619^*^	7.538 ± 1.560^*^
LOC106778742	21.863^*^	23.031^*^	4.944 ± 1.024^*^	1.160 ± 0.141
LOC106772062	34.174^*^	44.594^*^	3.126 ± 0.693^*^	0.340 ± 0.070^*^
LOC106772219	18.091^*^	21.205^*^	1.225 ± 0.203	1.453 ± 0.258^*^
LOC106761476	0.164^*^	0.424^*^	0.081 ± 0.015^*^	0.021 ± 0.005^*^
LOC106764964	43.542^*^	101.780^*^	9.171 ± 1.960^*^	5.646 ± 1.174^*^
LOC106777641	10.565^*^	24.433^*^	2.292 ± 0.228^*^	1.170 ± 0.238
LOC106757953	0.048^*^	0.182^*^	0.053 ± 0.005^*^	0.042 ± 0.005^*^
LOC106773632	3.548^*^	8.649^*^	3.175 ± 0.540^*^	1.256 ± 0.313
LOC106767260	41.569^*^	298.831^*^	12.767 ± 2.110^*^	2.703 ± 0.492^*^
LOC106763861	55.894^*^	124.501^*^	1.606 ± 0.216^*^	1.892 ± 0.327^*^
LOC106776083	6.184^*^	2.457^*^	2.787 ± 0.339^*^	1.622 ± 0.354^*^
LOC106766799	23.465^*^	62.532^*^	20.318 ± 3.213^*^	9.806 ± 1.580^*^
LOC106766798	0.510	0.469^*^	0.227 ± 0.017^*^	0.147 ± 0.027^*^

Asterisk (*) means gene with deferential expression by RNA sequencing in RPKM units or significant differences. The qRT-PCR results were expressed as the average of three biological replicates with standard deviation.

## Discussion

In this study, we have used large-scale “omics” technologies to obtain an overview of transcriptome and metabolome reprogramming in response to mung bean post-germination seedling growth. These results revealed that induction and execution of seedling growth were the concerto of a number of cellular and biochemical processes.

Catabolic pathways were induced early upon imbibition to sustain germination and seedling growth. The stored macromolecules, such as carbohydrates, proteins, and lipids, were degraded into micromolecules, including amino acids, monosaccharides, and lipid metabolites, to produce the energy needed for the physiological activities^23^. The changes in the transcripts and metabolites profiles revealed the activation of primary metabolism during mung bean post-germination seedling growth (Fig. 3E and Fig. 4).

Here, mung bean germination led to increased levels of sucrose and monosaccharides including glucose and fructose, which entered into the glycolysis and TCA cycle to produce ATP. The further metabolites that were metabolized by other pathways, such as aromatic amino acids (Phe and Tyr), in combination with various amines, were the primary precursors for the induced hydroxycinnamic acid biosynthesis leading to accumulation of health-promoting phenolic compounds. Furthermore, sugars can also cross-talk with various phytohormones signalling networks and other factors, which modulate critical growth processes such as seed germination and seedling growth^24^.

Upon imbibition, carbohydrate metabolism was activated immediately to produce energy and sustain axis growth. Similar results were also shown in soybean, rice and barley germination, with transcripts encoding components involved in starch and sucrose, glycolysis, and TCA cycle being upregulated from 6-HAI to 3-DAI^21,25,26^, following by a relative stable level. At the same time, the accumulation of monosaccharides and consumption of raffinose and citrate also revealed similarities with metabolomic analysis of soybean during germination^27^.

Mung bean is a potential source of most essential amino acids with the exception of sulphur-containing amino acids including methionine and cysteine^28^. The profiles of most amino acids during mung bean germination were similar to the results reported previously^11^, although there were some variations among the reported values of the amino acid contents, which might be explained by the differences in mung bean varieties and analytical methods^29^.

After 6-h imbibition, methionine was observed, and then was accumulated significantly till 6-day growth (Fig. 4D). Other essential amino acids were also exhibited dramatic increasing trends during mung bean post-germination seedling growth. These amino acids along with the mono- and oligosaccharides changed during post-germination seedling growth are also positively contributed to health benefits and the unique taste of mung bean sprouts^30^. Previous studies indicated that amino acids used for *de novo* protein synthesis came from the degradation of storage proteins in several species (e.g. barley)^31^. Here, 50 out of 70 DEGs involved in amino acid synthesis were upregulated from 6-HAI to 3-DAI, and 11 out of 20 DEGs were increased from 3-DAI to 6-DAI in this study. The results indicated that the accumulation of amino acids during mung bean post-germination seedling growth was partly attributed to activation of some amino acid biosynthesis pathways at early stage of seedling growth, as reported in soy bean sprouts^21^. Furthermore, all the essential amino acids for human health were accumulated extensively in mung bean sprouts at 6-DAI, indicating that mung bean sprouts might be a good source of amino acids.

Plant hormones, as internal mediators of developmental and environmental factors, play an important role in mung bean germination. ABA and GA are considered as major players in the regulation of plant development including regulation of seed maturation, germination inhibition and reduction of stomatal aperture^32,33^. The amounts of endogenous plant hormones are regulated by the balance between *de novo* synthesis and catabolism. Previous studies have proposed that NCED and CYP707A are the main enzymes regulating biosynthesis and catabolism of ABA, respectively^34^. Thus, germination would be preceded by a lower amount of ABA resulting from suppressed NECD and activated CYP707A after imbibition (Fig. 5, Table S8). After that, the accumulation of ABA at 6-DAI might be explained by the response to water stress of newly born root, which induced ABA biosynthesis for translocation to the apoplast surrounding leaf guar d cells by the reduction of stomatal aperture and evapotranspiration^35^. In contrast, the decrease of GA3 might be the results of down-regulated by *ent*-copalyl diphosphate synthase (CPS), *ent*-kaurene synthase (KS), *ent*-kaurene oxidase (KO) and *ent*-kaurenoic acid hydroxylase (KAO) genes (Fig. 5, Table S8). Additionally, the growth and elongation of root at later stage of germination might be the results of the regulation of IAA and GA^36^.

JA, synthesized from 𝛼-linolenic acid metabolism pathway^37^, regulates diverse developmental processes and defence response^38^. The increase of JA might be the results of the induction of biosynthesis during germination and seedling growth. The DEGs encoding allene oxide cyclase (AOC), acyl-CoA oxidase (ACX) and enoyl-CoA hydratase/3-hydroxyacyl-CoA dehydrogenase (MFP2) were all up-regulated at the early stage of germination (Fig. 5, Table S8). The lower level of JA in the 6-HAI might be associated with its germination-inhibiting role, as reported in Arabidopsis seeds (non-dormant Columbia seeds) after imbibition^39^. Intriguingly, all the JA response DEGs were observed from 6-HAI to 3-DAI (Table S8), suggesting JA signalling was more active in responsive to early growth stage of mung bean sprouts.

IAA is the major naturally occurring auxin, which involved in regulation of plant growth and development through inducing cell elongation and cell division^40^. During mung bean post-germination seedling growth, the numbers of auxin signalling DEGs exceeded the number of DEGs involved in other hormones signalling (Table S8). Although the role of IAA in seed germination is not necessary, IAA is present in the seed radicle tip during and after seed germination for the growth of young seedlings^9^. The lowest contents of IAA at 3-DAI might be the supplement to axes from cotyledons, which was similar with *Phaseolus vulgaris* and *Arabidopsis thaliana*^39,41^.

Except for ABA, GA, JA and IAA, other plant hormones such as cytokinin, brassinosteroid, ethylene and salicylic acid were also responsive to mung bean sprouts growth (Table S8). Brassinosteroid and ethylene were found to enhance effects on seed germination^9^. Salicylic acid, known as response to biotic and abiotic stress, also involves in the regulation of physiological and chemical process in higher plants. The role of exogenous SA in germination depends on the plant species and the dosages employed, which could inhibit germination or increase seed vigour^42^. The profiles of DEGs involved phytohormone signalling during mung bean germination provided considerable insight into the complexity of those signalling cascades. The numbers of DEGs involved in hormone signalling at the early stage exceeded those at the later stage, which might be explained by more prominent physiological changes during the early seedling growth stage, such as emergence of radicle and hypocotyl.

Furthermore, phytohormones regulate germination not only via single pathways, but through complex interactions of various signalling pathways^9,43^. All the plant hormones form an interlocked signalling network and interact with one another to finely control germination, including in response to environmental constraints. Hopefully, the transcripts and metabolites profiles might contribute to elucidate the roles of hormones in the germination and seedling growth.

Seed germination is a sophisticated process and involves the concerted action and interaction of a wide number of genes. Primary metabolites including carbohydrates and amino acids changed dramatically, along with alteration of plant hormones. At the early stage (6-HAI to 3-DAI), rapid changes in transcript level occurred, including starch and sucrose metabolism, glycolysis, citrate cycle, biosynthesis of amino acids, and plant hormone metabolism and signal transduction. Later changes in the metabolites (3-DAI to 6-DAI), including carbohydrates and amino acids, appeared to be driven by rearrangement of the transcriptome. The process was also accompanied with changes in hormones and their signal transduction. This study was the first comprehensive study using integrated transcriptomic and metabolomic techniques to investigate the process of mung bean post-germination seedling growth. These results will provide insights into molecular mechanisms underlying the metabolic regulation during mung bean post-germination seedling growth, suggesting that germination might be potential process to improve mung bean nutritional quality.

## Methods

### Sample preparation

Mung bean (*Vigna radiata*) seeds were obtained from Shanxi Academy of Agricultural Sciences (Taiyuan, China). Selected seeds were sterilized in 75% ethanol for less than 1 min, soaked in distilled water for 6 h at room temperature (20 °C). Then the seeds were placed into the sprouter (150 cm × 180 cm), and kept in the dark at 20 °C for germination. The seeds were rinsed with distilled water twice a day. Three biological replicates were treated in this study. Samples were collected at 6-hour, 3-day, and 6-day after imbibition and stored at −80 °C for further analysis.

### RNA isolation and sequencing

Total RNA was isolated by Plant RNA Extraction Kit (TransGen Biotech Co., Ltd., China). RNA quality was assessed using 1% agarose gel, Nano Photometer spectrophotometer (IMPLEN, CA, USA) and Agilent Bioanalyzer 2100 system (Agilent Technologies, CA, USA). Then the total RNA was provided to Beijing Biomarker Technologies Inc. (Beijing, China) for transcriptome sequencing. In brief, the total RNA was used to enrich mRNA using Poly-T oligo-attached magnetic beads. Then the purified mRNA was fragmented and constructed into cDNA library. Followed by purifying the PCR products (AMPure XP system), library quality was assessed on the Agilent Bioanalyzer 2100 system (Agilent, Santa Clara, CA, USA) and then sequenced on an Illumina Hiseq. 2500 platform (Illumina, San Diego, CA, USA).

### RNA-Sequencing data analysis

Raw data (raw reads) were cleaned by discarding reads containing adapter, reads containing Ploy-N and low quality reads(q) < 20 from raw data. These cleaned reads were then mapped to the reference genome sequence (https://www.ncbi.nlm.nih.gov/genome/?term=mung+bean) using Tophat2. Quantification of gene expression levels were calculated as: fragments per kilobase of transcript per million fragments mapped (FPKM) = $\frac{{\bf{c}}{\bf{D}}{\bf{N}}{\bf{A}}\,{\bf{F}}{\bf{r}}{\bf{a}}{\bf{g}}{\bf{m}}{\bf{e}}{\bf{n}}{\bf{t}}{\bf{s}}}{{\bf{M}}{\bf{a}}{\bf{p}}{\bf{p}}{\bf{e}}{\bf{d}}\,{\bf{F}}{\bf{r}}{\bf{a}}{\bf{g}}{\bf{m}}{\bf{e}}{\bf{n}}{\bf{t}}{\bf{s}}\,({\bf{M}}{\bf{i}}{\bf{l}}{\bf{l}}{\bf{i}}{\bf{o}}{\bf{n}}{\bf{s}})\times {\bf{T}}{\bf{r}}{\bf{a}}{\bf{n}}{\bf{s}}{\bf{c}}{\bf{r}}{\bf{i}}{\bf{p}}{\bf{t}}\,{\bf{L}}{\bf{e}}{\bf{n}}{\bf{g}}{\bf{t}}{\bf{h}}\,({\bf{k}}{\bf{b}})}$.Genes with an adjusted fold change (FC) ≥ 2 and false discovery rate (FDR) ≤ 0.01 found by DESeq were assigned as differentially expressed. The DEGs were subjected to KEGG ontology enrichment analysis using KOBAS software^44^.

### Metabolic profiling of mung bean by GC-MS

The metabolome of mung bean sample was extracted with a mixed solution (methanol: chloroform: water = 5: 2: 2) according to the method described in our previous study^45^. Each group (6-HAI, 3-DAI and 6-DAI) contained 6 replicate samples. The extract was dried in a speed vacuum concentrator, and derivatized with N-methyl-N-(trimethylsilyl) trifluoroacetamide (MSTFA) following the protocol described before^46^. 1 µL for each sample was injected into a DB-35MS UI capillary column (30 m × 0.25 mm, 0.25 μm, Agilent) at 280 °C in split mode (50: 1) with helium carrier gas flow set to 1 mL/min. The temperature was isothermal for 5 min at 85 °C, followed by 8 °C per min ramp to 205 °C, held at this temperature for 5 min, and then ramped at 8 °C per min to 300 °C, held for 5 min. The mass range was from m/z 60 to 1000. The temperature of transfer line and ion source were set according to the previous study^46^. GC-MS data analysis was conducted using Chroma TOF 4.3X software of LECO Corporation (Saint Joseph, MI, USA). The metabolites were annotated based on LECO-Fiehn Rtx5 database, NIST library, and in-house database. The multivariate statistical analyses of the metabolome data were performed using SIMCA software package (V14, Umetrics AB, Umea, Sweden).

### Endogenous hormone quantification by liquid chromatography tandem mass spectrometry (LC-MS)

The endogenous hormones in mung bean sample were extracted according to Pan *et al*.^47^, and separated using a Shimadzu ultra-fast LC-20A system equipped with C18 column (AQUITY UPLC BEH 130, 2.1×100 mm, 1.7 μm, Waters) as previous reported^48^. The mobile phase consisted of 5 mM ammonium formate and 0.1% formic acid dissolved in water (A) and 5 mM ammonium formate and 0.1% formic acid dissolved in methanol (B) using a gradient elution of 30% B at 0–2 min, 30–100% B at 2–20 min, 100% B at 20–22 min, 100–30% B at 22–25 min, and 70% B at 25–30 min. The flow rate was set at 0.1 mL/min, and the column was maintained at 30 °C. The eluate was subsequently introduced into the electrospray ion source of a tandem triple quadrupole MS analyser (API4000, AB SCIEX, Foster City, CA, USA), and the 11 hormone compounds were quantified in multiple reaction monitoring (MRM) mode using optimized MS/MS conditions, which were listed in Table S9. The MS conditions were set with the same detailed MS parameters described in our previous study^48^. The Analyst 1.5.2 software (AB SCIEX, Foster City, CA, USA) was used to control the instrument and to acquire and process all of the MS data. The amounts of 11 plant hormones in the samples were determined using external standards except IAA and SA, which were quantified using internal standards d5-IAA (indol-3-acetic-2,2-d_2_ acid; Sigma Aldrich, CAT. No. 492817) and d6-SA (2-hydroxybenzoic acid-d_6_; Sigma Aldrich, CAT. NO. 616796) respectively. Specifically, for the external standard method, we calculated the concentration of hormones followed its authentic standard curves. The hormone amounts were then normalized to the mass of fresh plant tissue determined by weighing before extraction. For the internal standard method, we quantified the two hormones by calculating the ‘response factor’ of each authentic hormone in comparison with its corresponding internal standard as described in the previous study^47^. The hormone amounts were then normalized to the mass of fresh plant tissue determined by weighing before extraction.

### Gene expression study by qRT-PCR

The qRT-PCR for gene expression was performed in duplicate using a ChamQ SYBR Color qPCR Master Mix (Vazyme Biotech Co., Ltd., China) on a CFX384 Touch Real-Time PCR Detection System (Bio-Rad, Hercules, CA, USA). The specificity of the amplification was confirmed by the melting curve. The primers were designed using Primer Premier 5.0 software (Premier, Palo Alto CA, USA) and were synthesized by Generay Biotech Co., Ltd. (Shanghai, China). The primers of target genes were listed in Table S10. Threshold cycle (C_t_) values were analysed using Bio-Rad CFX Manager software. The relative expression of the target genes was calculated using the 2^−ΔΔCt^ method and reported as mean ± SD (n = 3).

## Supplementary information

Supplementary information.

Supplementary information 2.

Supplementary information 3.

## Data Availability

The datasets generated for this study can be found in the NCBI SRA database and the SRA accession number is PRJNA546066 (https://www.ncbi.nlm.nih.gov/sra/PRJNA546066).
