# Supplementary material for: Integrated Transcriptomic and Metabolic Framework for Carbon Metabolism and Plant Hormones Regulation in Vigna radiata during Post-Germination Seedling Growth
Source: Sci Rep. 2020 Feb 28;10:3745. doi: 10.1038/s41598-020-60771-3 (PMC7048927; doi:10.1038/s41598-020-60771-3)
Supplement: Supplementary file 1 — Supplementary information. [file 41598_2020_60771_MOESM1_ESM.docx]

**Integrated Transcriptomic and Metabolic Framework for Carbon Metabolism and Plant Hormones Regulation in *Vigna radiata* during Post-Germination Seedling Growth**

Hong Wang^1^, Xinbo Guo^1, *^_,_ Quan Li^1^, Yanyan Lu^1^, Wenjie Huang^2^, Fangyuan Zhang^3^, Ling Chen^1^, Rui Hai Liu^4^, ShijuanYan^2, *^

^1^School of Food Science and Engineering, Ministry of Education Engineering Research Centre of Starch & Protein Processing, Guangdong Province Key Laboratory for Green Processing of Natural Products and Product Safety, South China University of Technology, Guangzhou, 510640, China

^2^Agro-biological Gene Research Center, Guangdong Academy of Agricultural Sciences, Guangzhou, 510640, China

^3^School of Life Science, Southwest University, Chongqing, 400715, China

^4^Department of Food Science, Stocking Hall, Cornell University, Ithaca, NY, 14853, USA

***Correspondence:**Xinbo Guo (email: guoxinbo@scut.edu.cn)

Shijuan Yan (email: shijuan@agrogene.ac.cn)

# Supplementary Information

**Supplementary Figure 1** The overview of gene expression of samples from mung bean post-germination seedling growth. (a) Heat map of correlation coefficient values among samples. Gradient color barcode at the left indicates the minimum vale in pink and the maximum in green. Cluster tree were built beside Heat map to indicate the relationship of samples. (b) Global transcriptional pattern of mung bean and sprouts during germination. FPKM values of expressed genes（FPKM>1） were converted to z-scores and color intensities range from -1 to 1. (Word file)

**Table S1** Alignments of RNA-Sequencing reads on the mung bean genome. (Word file)

**Table S2** Functional annotation of new unigenes in mung bean. (Word file)

**Table S3** Differentially expressed genes between at least two seedling growth points in mung bean sprouts (Excel file).

**Table S4** KEGG classification of DEGs between sequential growth time points (Excel file).

**Table S5** Identified metabolites of samples from mung bean post-germination seedling growth (Excel file).

**Table S6** DEGs involved in primary metabolism represented in Figure 4 (Excel file).

**Table S7** The contents (ng/100 mg) of common plant hormones in mung bean and sprouts during post-germination seedling growth. (Word file)

**Table S8** DEGs involved in plant hormone metabolism and signal transduction represented in Figure 5 (Excel file).

**Table S9** Characteristic fragment ions of the hormone standards and their optimized MS/MS conditions. (Word file)

**Table S10** Primers used in real-time RT-PCR. (Word file)
