# Supplementary material for: Integrated Transcriptomic and Metabolic Framework for Carbon Metabolism and Plant Hormones Regulation in Vigna radiata during Post-Germination Seedling Growth
Source: Sci Rep. 2020 Feb 28;10:3745. doi: 10.1038/s41598-020-60771-3 (PMC7048927; doi:10.1038/s41598-020-60771-3)
Supplement: Supplementary file 2 — Supplementary information 2. [file 41598_2020_60771_MOESM2_ESM.docx]

## Supplementary Figures


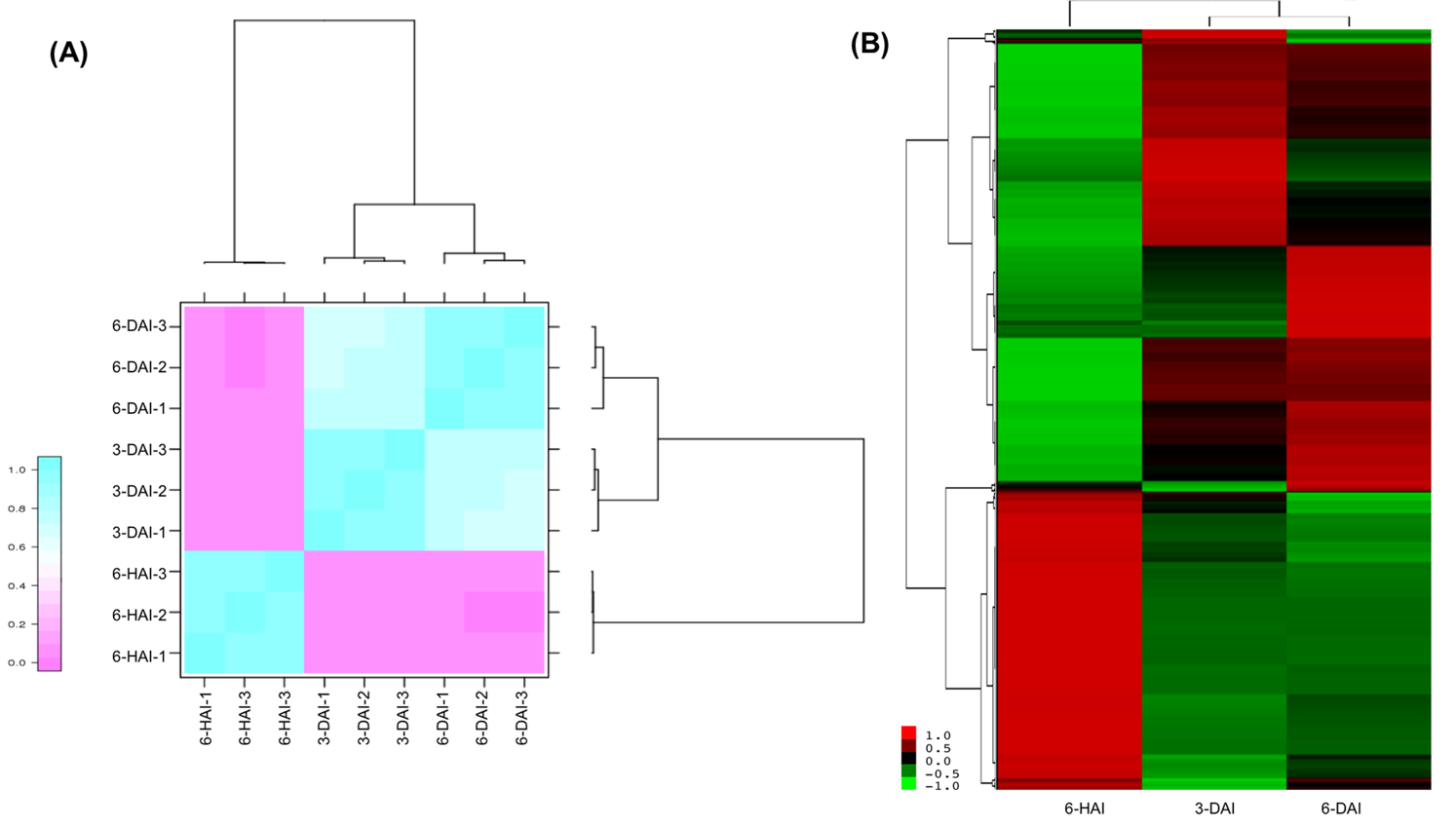


**Supplementary Figure 1.** The overview of gene expression of samples from mung bean post-germination seedling growth. (A) Heatmap of correlation coefficient values among samples. Gradient color barcode at the left indicates the minimum vale in pink and the maximum in green. Cluster tree were built beside Heat map to indicate the relationship of samples. (B) Global transcriptional pattern of mung bean and sprouts during germination. FPKM values of expressed genes（FPKM>1） were converted to z-scores and color intensities range from -1 to 1.

## Supplementary Tables

**Table S1** Alignments of RNA-sequencing reads on the mung bean genome

| Germination stage | Total reads | Mapped reads | Uniquely mapped | Multiple matches |
| --- | --- | --- | --- | --- |
| 6-HAI | 34,554,399 | 27,191,414 | 26,741,443 | 449,971 |
| 3-DAI | 39,201,211 | 32,879,565 | 32,400,733 | 478,832 |
| 6-HAI | 41,054,716 | 34,277,378 | 33,249,484 | 1,027,894 |

**Table S2** Functional annotation of new unigenes in mung bean

| Database | GO | KEGG | SwissProt | eggNOG | Nr | All |
| --- | --- | --- | --- | --- | --- | --- |
| Annotated Number | 385 | 263 | 430 | 704 | 808 | 812 |

**Table S7** The contents (ng/100 mg) of common plant hormones in mung bean and sprouts during post-germination seedling growth^*^

| Hormones | 6-HAI | 3-DAI | 6-DAI |
| --- | --- | --- | --- |
| gibberellin A4 (GA4) | 0.192±0.001 | 0.203±0.023 | 0.193±0.001 |
| indole-3-butyric (IBA) | 0.150±0.007 | 0.154±0.007 | 0.152±0.008 |
| salicylic acid (SA) | 84.81±6.54c | 4.36±0.93a | 12.98±2.5b |
| zeatin riboside (ZR) | 0.605±0.000b | 0.603±0.001a | 0.606±0.001b |
| cinnamic acid （CA） | 0.121±0.082a | 0.619±0.206c | 0.380±0.100b |
| jasmonic acid methyl ester (MeJA) | nd | nd | 1.12±0.41 |
| zeatin | nd | nd | nd |

^*^Values (mean ± SD) in the same row with different letters differ significantly at p <0.05. “nd” means “not detect”.

**Table S9** Characteristic fragment ions of the hormone standards and their optimized MS/MS conditions.

| Analyte | ESI mode | Transition1 | | | Transition2 | | |
| --- | --- | --- | --- | --- | --- | --- | --- |
|  |  | Quantitative ion | Collision energy/ev | Declustering potential/ev | Quantitative ion | Collision energy/ev | Declustering potential/ev |
| ABA | ESI- | 262.9>152.8 | -15 | -60 | 262.9>203.9 | -25 | -60 |
| GA3 | ESI- | 345>142.8 | -40 | -75 | 345>220.9 | -33 | -75 |
| JA | ESI- | 209>58.8 | -25 | -60 | 209>164.8 | -17 | -60 |
| IAA | ESI+ | 176.1>77.1 | 60 | 58 | 176.1>130.1 | 20 | 58 |
| GA4 | ESI- | 331>257.1 | -32 | -96 | 331>212.9 | -42 | -96 |
| IBA | ESI- | 201.9>115.8 | -22 | -90 | 201.9>157.8 | -20 | -90 |
| SA | ESI- | 136.9>65.0 | -40 | -40 | 136.9>92.8 | -32 | -40 |
| ZR | ESI- | 350.1>217.8 | -20 | -39 | 350.1>199.8 | -40 | -39 |
| CA | ESI- | 147>102.9 | -22 | -60 | 147>77 | -30 | -60 |
| MeJA | ESI+ | 225.1>105 | 35 | 62 | 225.1>67 | 50 | 62 |
| Zeatin | ESI+ | 220.2>136.1 | 24 | 64 | 220.2>148.1 | 20 | 64 |

**Table S10** Primers used in real-time RT-PCR

| **Gene ID** | **Primer sequence（5’—3’）** |
| --- | --- |
| LOC106771984 | F: ACCACAGCTGAGCGAGAAAT |
|  | R: ATCATGGATGGCTGGAAGAG |
| LOC106767203 | F: ACTGGTGGTTGTAGGTGTGT |
|  | R: ATCAAAGGTAGCACAGCCAC |
| LOC106753951 | F: TTCACGGCCAAACAGAGTTG |
|  | R: TTGGTTTTGGAGCCTTTGGG |
| LOC106760472 | F: TCTTCCTCCATGCGTCCTTT |
|  | R: GTCGCTGATGGGTCCTATGA |
| LOC106754175 | F: GGTGCCATGGAATACAGCTG |
|  | R: GGAACAAACACCACCTTCCC |
| LOC106772114 | F: AAAGCCACCTCAATGCTTGG |
|  | R: GATGTTGGGCTTGGTTCCTC |
| LOC106755252 | F: AAGCCTGAGTTCACCGATCA |
|  | R: TTTCGTGCAGTTTCTTGGCA |
| LOC106778742 | F: CCTACCCCACACTGCAACTA |
|  | R: GGCAAACATGGAGTGACGTT |
| LOC106772062 | F: TGCTTCGTTCAACCTTTGCA |
|  | R: GGGTTACGGTACTCCTCCAC |
| LOC106772219 | F: ACACTGCTTGGTACTTGGCT |
|  | R: AACTGCATGACCACCAAACG |
| LOC106761476 | F: TTAGGTCTTGCTCTCCCCAC |
|  | R: AGTTGCGCTTGTGTTTGACA |
| LOC106764964 | F: ACAAATGCTCCTGGTTTGCA |
|  | R: ATCAGGGAATGCTTGGCTCT |
| LOC106777641 | F: TAAGCAGCAACTCCAGTCCA |
|  | R: ATGACATGATGGTGCACGTG |
| LOC106757953 | F: GTCCAAACGAACCATCCGAG |
|  | R: ACTGCCGCACATTTGTTGAT |
| LOC106773632 | F: GTTGTTGGTCAGGATCAGCC |
|  | R: TGGAGACTTCACCCATTCCC |
| LOC106767260 | F: AAGGGCGTGGAGGAGTTTTA |
|  | R: ATCCACCACCTCATTCAGCA |
| LOC106763861 | F: GGTTCCATTCATCCACGACA |
|  | R: AGAGACGAAAAGCCACTCCA |
| LOC106776083 | F: GGTGGTTTCATTGCCTCACA |
|  | R: AGGTGGAACTCATGGCAGAA |
| LOC106766799 | F: AGCCTGGCCTAGAAGAAGTG |
|  | R: ACCAGCTCCGATTCCTTTGA |
| LOC106766798 | F: AGCTGGGTATGTTGGAGGAC |
|  | R: CGGCTCATAGATCGGAAGGT |
